# Taxonomic revision of the genus *Mesechinus* (Mammalia: Erinaceidae) with description of a new species

**DOI:** 10.24272/j.issn.2095-8137.2018.034

**Published:** 2018-04-25

**Authors:** Huai-Sen Ai, Kai He, Zhong-Zheng Chen, Jia-Qi Li, Tao Wan, Quan Li, Wen-Hui Nie, Jin-Huan Wang, Wei-Ting Su, Xue-Long Jiang

**Affiliations:** 1 Gaoligongshan National Nature Reserve, Baoshan Yunnan 678000, China; 2 Kunming Institute of Zoology, Chinese Academy of Sciences, Kunming Yunnan 650223, China; 3 The Kyoto University Museum, Kyoto University, Kyoto 606-8501, Japan; 4 College of Life Sciences, Anhui Normal University, Wuhu Anhui 241000, China; 5 Nanjing Institute of Environmental Sciences, Ministry of Environmental Protection, Nanjing Jiangsu 210042, China

**Keywords:** *Mesechinus*, Taxonomy, Morphometrics, Inhibitory cascade, Karyotype, New species, Supernumerary molar

## Abstract

Hedgehogs in the genus *Mesechinus* (Family Erinaceidae), which include two currently recognized species (*M. dauuricus* and *M. hughi*), are distributed from northeast Mongolia to the upper Amur Basin in Russia and adjacent areas in northeast and northern China. In recent years, a population of *Mesechinus* hedgehogs was discovered from Mt. Gaoligong, southwestern Yunnan, China, far from the known distribution range of the genus. Furthermore, these hedgehogs are the only known population to be distributed at elevations higher than 2 100 m and in sympatry with gymnures. To evaluate the taxonomic status of these hedgehogs, we examined specimens representing *Mesechinus* taxa in China and further conducted morphometric and karyotypic analyses. Our results supported the existence of four species in China. Specifically, we identified the hedgehogs from Mt. Gaoligong as a new species, *Mesechinus wangi* **sp. nov.**, and recognized *M. miodon*, previously considered as a synonym of either *M. dauuricus* or *M. hughi*, as a distinct species. Interestingly, we observed a supernumerary M^4^ on all specimens of *Mesechinus wangi* **sp. nov.**, which is an extremely rare event in the evolution of mammalian dentition.

## INTRODUCTION

**Figure 1:**
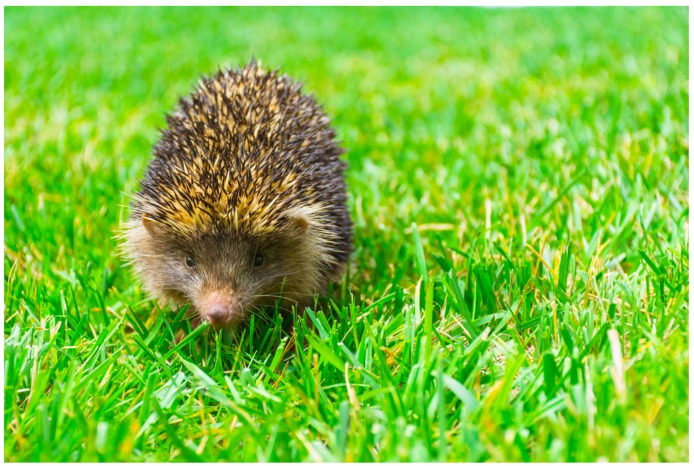
Living *Mesechinus* wangi sp. nov. (KIZ 034115)

Extant erinaceids, including spiny hedgehogs (Erinaceinae) and silky-skinned gymnures and moonrats (Galericinae), are found within the family Erinaceidae (Hutterer, 2005). The monophyly of each subfamily, as well as their sister-relationships, are well supported in various morphological and molecular studies (Corbet, 1988; Frost et al., 1991; He et al., 2012). These molecular studies also suggest that the living gymnures diverged from the ancestor of hedgehogs 40 million years ago (Ma), which is far older than the most recent common ancestor of living hedgehogs (Bannikova et al., 2014). Members in the two subfamilies are not only morphologically and genetically distinct but also characterized by different geographic distributions and habitats (Corbet, 1988). The living species in Galericinae are mainly distributed in humid montane forests of subtropical and tropical Southeast Asia (*Echinosorex*, *Hylomys* and *Podogymnura*), Southern China (*Hylomys* and *Neotetracus*), and Hainan Island (*Neohylomys*). With their most recent common ancestor considered to be in the late Miocene (Bannikova et al., 2014), living hedgehogs have adapted to diverse habitats and are widely distributed throughout Africa (*Atelerix* and *Paraechinus*) and Eurasia (*Erinaceus*, *Hemiechinus*, *Mesechinus* and *Paraechinus*) in deciduous woodland, coniferous forest, forest steppe, grasslands, savanna, dry steppes, semi-desert, and even arid desert (Corbet & Hill, 1992); until recently, however, they have never been found in tropical or subtropical rainforest. In 2003, Ai (2007) discovered a small population of hedgehogs from the southern-most edge of Mt. Gaoligong in Yunnan Province at approximately 2 200–2 600 m a.s.l., near the border between China and Myanmar. These hedgehogs are characterized by the absence of a spineless section on their head and by ears of similar length to the surrounding spines, suggesting that they are members of the genus *Mesechinus* (Figure 1). The discovery was unexpected and of interest because: (1) the location is at least 1 000 km from the known distribution of any other hedgehog species; (2) the elevations are higher than that of any known hedgehog habitat; (3) the habitat is subtropical montane evergreen broad-leaved forest, which is typical habitat of the gymnures but differs from any known hedgehog habitat; and (4) the animals are sympatrically distributed with gymnures (*Neotetracus sinensis*), which is also the first ever record. While these clues indicate that the population represents a distinct taxon, its taxonomic status has yet to be resolved.

**Figure 2:**
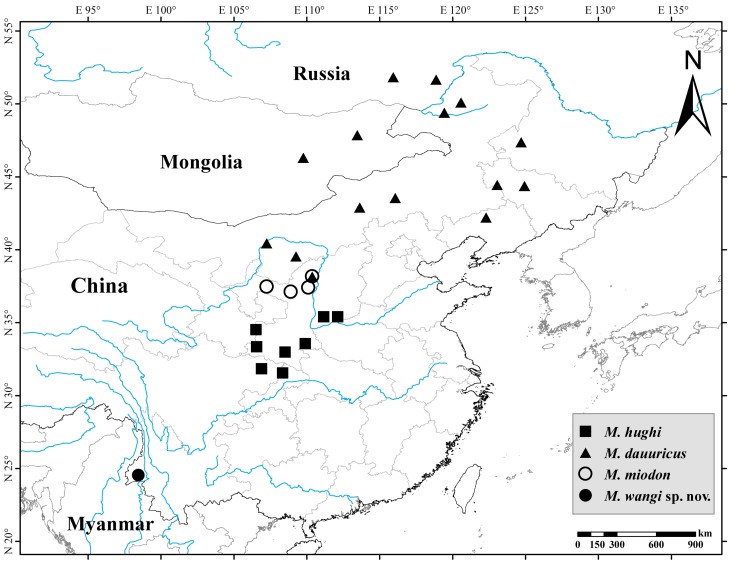
Distribution of genus *Mesechinus*

*Mesechinus* hedgehogs are mainly distributed in northern China and Mongolia, as well as the Transbaikalia region and upper Amur Basin in Russia (Figure 2). Two species (*M. dauuricus* and *M. hughi*) were recognized in Mammal Species of the World (Hutterer, 2005). After Sundevall described the type species *Erinaceus* (*Mesechinus*) *dauuricus* in 1842, another five forms were recognized, including *przewalskii* Satunin, 1907, *hughi* Thomas, 1908, *miodon* Thomas, 1908, *manchuricus* Mori, 1927, and *sylvaticus* Ma, 1964. Subsequently, however, *manchuricus*, *przewalskii*, and *sibiricus* were recognized as synonyms of *M. dauuricus* (Corbet, 1988), and *miodon* and *sylvaticus* as synonyms of *M. hughi*. The most debated species continues to be *miodon*, which was originally described together with *hughi* by Thomas (1908). Based on successive morphological research, some authors have included it in *M. dauuricus* (Corbet, 1978; Corbet & Hill, 1992), whereas others have included it in *M. hughi* (Hoffmann & Lunde, 2008; Hutterer, 2005). Furthermore, karyotypic study of *miodon* from its type locality demonstrated variable chromosomal numbers ranging from 2*n*=44 to 48 (Lin & Min, 1989; Kong et al., 2016a) due to the existence of B-chromosomes (Kong et al., 2016a), which has been interpreted as evidence of full species status (Kong et al., 2016a, 2016b). However, B-chromosomes are rarely used for delimiting species and as its craniodental morphology has not yet been fully diagnosed, the species status of *M. miodon* remains suspicious.

In this study, we integrated morphometric and karyotypic approaches to revisit the taxonomy of *Mesechinus*. We examined whether *M. miodon* is distinguishable from other species and were particularly interested in the taxonomic status of the hedgehog population found from Mt. Gaoligong.

## MATERIALS AND METHODS

### Specimens examined

We examined 59 specimens (skins and skulls) of *Mesechinus* deposited in the Institute of Zoology (IOZ) and Kunming Institute of Zoology (KIZ) of the Chinese Academy of Sciences (CAS), Shaanxi Institute of Zoology (SXIZ), Northwest University (NWU), and China West Normal University (CWNU) (see Supplementary Appendix I). These specimens included the *M. hughi sylvaticus* holotype. Photo images of the *M. miodon* holotype were also obtained for examining diagnosable characters and for morphological description and comparison with other named species. Morphology was examined and described following Corbet (1988), Frost et al. (1991), Gould (1995), and Thomas (1908). Based on our diagnosis and comparison of external and craniodental morphology, we recognized four species/putative species, including *M. dauuricus* (to include *M. dauuricus dauuricus* (*n*=8) and *M. dauuricus manchuricus* (*n*=5)), *M. hughi* (to include *M. hughi hughi* (*n*=28) and *M. hughi sylvaticus* (*n*=3)), and *M. miodon* (*n*=9). We recognized the animals from Mt. Gaoligong as a new species, which we name herein as *Mesechinus wangi* **sp. nov.** (*n*=6).

### Morphological measurement and analysis

External measurements, including body weight (W), head and body length (HB), tail length (TL), length of hind foot (HF), and ear length (EL), were recorded from specimen tags. Spine length (SL) was measured from specimens. Twelve cranial characters were measured in millimeters with a digital caliper graduated to 0.01 mm (Table 1) following Pan et al. (2007): greatest length of skull (GLS), condylobasal length (CBL), basal length (BL), cranial height (CH), palatal length (PL), zygomatic breadth (ZMB), interorbital breadth (IOB), mastoid width (MTW), greatest width of nasal (GWN), breadth of first upper molar (BM^1^), length of upper tooth row (LUTR), and length of below tooth row (LBTR). We extracted measurements from Allen (1938) for the eight specimens of *M. miodon* deposited in the Natural History Museum.

**Table 1:** External and cranial measurements (mm) of *Mesechinus* specimens examined in this study (mean±*SD* and range for each measurement and numbers of specimens measured (*n*) are given)

	*Mesechinus wangi*	*Mesechinus miodon*	*Mesechinus hughi*	*Mesechinus dauuricus*
	*n*=6	*n*=18*	*n*=31	*n*=13
W	411.20±48.66	505.00±168.73	341.39±127.82	562.41±130.37
	336.00–451.00; 5	230.00–750.00; 6	112.00–750.00; 31	423.00–840.00; 11
HB	202.40±26.10	195.22±24.26	189.71±24.20	206.21±22.30
	177.00–240.00; 5	120.00–220.00; 17	148.00–232.00; 31	175.00–261.00; 12
TL	17.26±1.82	33.22±5.22	19.23±3.32	24.08±3.65
	14.00–18.20; 5	25.00–43.00; 17	12.00–24.00; 27	17.00–30.30; 12
HF	47.20±1.20	58.80±85.13	37.97±4.36	34.74±7.39
	45.30–48.00; 5	35.00–378.00; 16	30.00–47.00; 31	18.00–41.00; 12
EL	29.60±1.74	28.81±3.13	22.94±3.99	31.19±3.44
	28.00–31.80; 5	24.00–34.50; 17	16.00–33.00; 31	22.30–34.00; 11
GLS	54.75±0.81	54.10±2.18	49.39±1.58	55.18±3.21
	53.70–55.60; 4	49.30–57.20; 14	45.10–52.40; 23	50.20–58.40; 12
CBL	54.55±0.68	53.18±2.47	48.46±1.61	54.72±2.94
	53.60–55.20; 4	48.50–56.30; 11	44.40–51.20; 23	49.40–57.40; 13
CH	17.13±0.69	18.67±0.72	16.14±0.97	18.37±0.58
	16.10–17.60; 4	17.80–19.70; 6	14.90–18.20; 21	17.20–19.10; 9
BL	50.00±1.58	49.64±2.12	45.55±1.32	51.83±2.02
	47.70–51.30; 4	44.70–52.30; 14	43.20–48.80; 21	48.10–54.50; 13
PL	30.25±0.58	28.82±1.46	26.58±0.63	28.60; 1
	29.50–30.80; 4	27.00–32.18; 14	25.70–28.40; 21	
ZMB	33.97±0.23	32.77±2.17	28.90±1.72	32.62±2.93
	33.70–34.10; 3	28.70–37.08; 14	25.70–32.00; 22	28.40–36.40; 13
IOB	14.68±0.38	13.87±0.83	12.51±0.52	13.86±0.72
	14.20–15.10; 4	12.90–15.10; 6	11.70–13.60; 23	13.00–15.10; 9
MTW	25.60±0.73	25.93±1.23	21.67±1.60	25.58; 1
	24.70–26.20; 4	24.30–28.30; 14	19.50–24.50; 21	
GWN	4.30±0.00	2.70±0.23	2.97±0.30	2.96; 1
	4.30–4.30; 3	2.37–2.94; 6	2.60–3.60; 23	
BM^1^	21.43±0.31	21.08±0.69	17.38±0.77	20.20; 1
	21.10–21.70; 3	20.30–22.30; 14	16.50–19.50; 21	
LUTR	27.90±1.18	27.25±1.03	24.65±1.15	27.85; 13
	26.70–29.10; 4	25.70–29.02; 14	21.40–26.10; 23	
LBTR	24.85±0.51	24.91±0.73	21.19±0.80	24.30; 1
	24.20–25.30; 4	23.40–25.70; 14	20.20–23.70; 21	

*: Includes measurement of nine specimens measured by Allen (1938). Abbreviations are given in the Materials and Methods section.

Morphometric variation was analyzed using principal component analysis (PCA) in SPSS v19.0 (SPSS Inc., Chicago, IL, USA). Only the 12 cranial measurements were used for PCA. All variables were log_10_-transformed before PCA. One-way analysis of variance (ANOVA) was used to test significant differences in external and cranial variables among species.

### Cell culture and cytological preparation

One specimen representing *Mesechinus wangi* **sp. nov.** (museum catalog number: KIZ 034115) was used for cell cultures. We followed Hungerford (1965) for cell culture and metaphase preparation. The fibroblast cell cultures derived from skin fibroblasts and bone marrow are stored in the Kunming Cell Bank, Kunming, Yunnan, China. Images were captured using the Genus System (Applied Imaging Corp., USA) with a CCD camera mounted on a Zeiss Axioplan 2 microscope. Chromosomes of *Mesechinus wangi* **sp. nov.** were arranged based on their relative length in order from longest to shortest.

## RESULTS

### Morphological comparison and diagnosis

**Figure 3:**
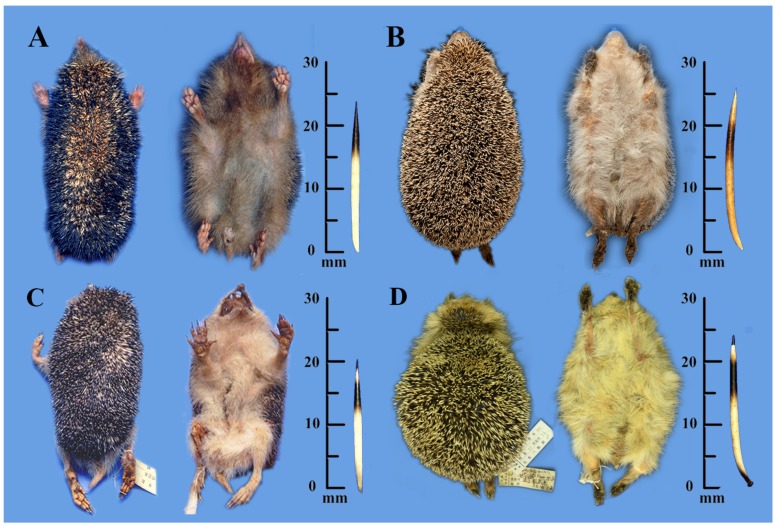
External morphs and spines of *Mesechinus wangi* sp. nov. (Type KIZ 022028) (A), *M. miodon* (Type BM 9.1.1.9) (B), *M. h. hughi* (KIZ 027029) (C), and *M. d. dauuricus* (KIZ 027005) (D)

**Figure 4:**
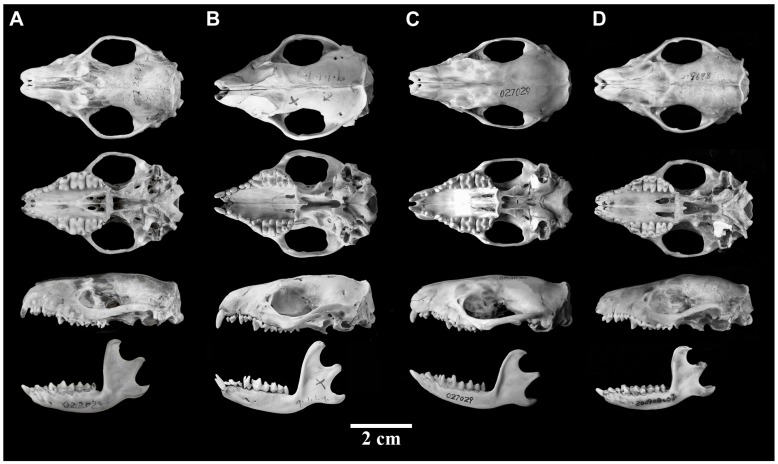
Dorsal ventral and lateral views of skull and mandible of *Mesechinus wangi* sp. nov. (Type KIZ 022028) (A), *M. miodon* (Type BM 9.1.1.9) (B), *M. h. hughi* (KIZ 027029) (C), and *M. d. dauuricus* (KIZ 027005) (D)

As mentioned previously, the hedgehogs from Mt. Gaoligong could be assigned to *Mesechinus* unambiguously based on external morphology. These animals lack a spineless area on their heads, which is distinct from *Atelerix*, *Erinaceus*, and *Paraechinus* (Figure 2, Figure 3), and their ears are similar to the surrounding spines in length, which is distinguishable from *Hemiechinus*. The skull and teeth are also characterized by several typical *Mesechinus* features, including a robust jugal reaching the lacrimal (Figure 4), shallow suprameatal fossa, and narrowly separated anterior and posterior borders of the suprameatal fossa (Figure 4), which distinguish it from all other genera (see Frost et al., 1991 for discussion). We compared the external and craniodental morphology of our specimens. It is worth noting that the sample size for some species/subspecies was small and may not reflect intra-specific variation, especially that of teeth (see discussion in Frost et al., 1991; Gould, 2001), which needs to be verified in future study.

All specimens examined in the current study showed few wholly white spines (Corbet, 1988; Figure 3). Spine lengths from longest to shortest were: *M. miodon* (~26 mm), *M. dauuricus* and *Mesechinus wangi* **sp. nov.** (~21–24 mm), and *M. hughi* (~21 mm). Spine color pattern was used by Thomas (1908) as a distinguishing feature for describing *M. hughi* and *M. miodon* (Figure 3). We found that *M. hughi* from Shaanxi (including topotype of *M. hughi hughi*) and Shanxi (holotype of *M. h. sylvaticus*) and *M. dauuricus* shared similar characters: that is, white for two-thirds of length, followed by black ring, narrow light ring, and black tip (Figure 3C, D). *Mesechinus miodon* was distinguished by spine light brown (rather than wholly white) for two-thirds of its length, followed by broad blackish-brown rings (rather than wholly black), light brown terminal (3–4 mm), and non-black tip (Figure 3D). *Mesechinus wangi* **sp. nov.** was differentiated from *M. hughi* by dark ring extending to tip on most spines, with narrow white ring near tip (Figure 3A).

Based on skull morphology, frontals were relatively higher than parietals in *M. hughi* and *Mesechinus wangi* **sp. nov.**, whereas parietals were higher than frontals in *M. dauuricus* and *M. miodon* (Figure 4). On the ventral side of the skulls, a posterior palatal shelf and well-developed spine were present on all specimens examined (Figure 4). *Mesechinus miodon* showed longer spines than other species (Figure 4B). An epipterygoid process was present in all specimens (Figure 4; Frost et al., 1991). In *M. miodon* this process was well developed, extending labially (Figure 4B), but only slightly or moderately developed in other taxa (Figure 4). The basisphenoid of *M. dauuricus* was previously considered to be uninflated, intermediate between the condition of *Hemiechinus* and that of *Atelerix* and *Erinaceus* (Frost et al., 1991). According to our examination, however, the basisphenoid was inflated in *M. dauuricus*, *M. hughi*, and *M. miodon* (Figure 4B, C), similar to the condition observed in *Hemiechinus auritus*, whereas the basisphenoid was uninflated in *Mesechinus wangi* **sp. nov.** (Figure 4A), similar to that of *Atelerix* and *Erinaceus*.

On the dorsal side of the skull, the nasal-maxilla relationship was used in Corbet (1988) and Ma (1964), though Frost et al. (1991) determined that the relationship exhibited too much inter-specific variation. Nevertheless, nasal breadth was obviously and significantly (see below) different between *Mesechinus wangi* **sp. nov.** and other species. More specifically, *Mesechinus wangi* **sp. nov.** was characterized by: nasal broad, premaxilla extending only slightly posteriorly and frontal extending only slightly anteriorly on dorsal side, premaxilla not touching frontal, and nasal and maxilla sharing long common sutures (Figure 4A). All other species exhibited much narrower nasal (Figure 4B–D). *Mesechinus hughi* could be characterized by: premaxilla extending posteriorly, frontal extending anteriorly, not touching premaxilla, with nasal and maxilla sharing short sutures (Figure 4C). *Mesechinus d*. *manchuricus* and *M. miodon* could be diagnosed by: premaxilla extending posteriorly, frontal extending anteriorly, premaxilla and frontal touching on dorsal side of skull (or nearly so), with nasal and maxilla not sharing common suture (Figure 4B, D).

**Figure 5:**
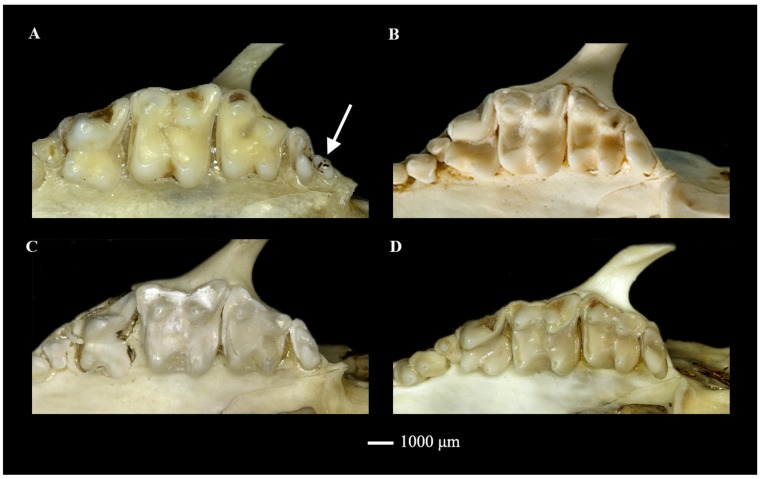
Right upper molars of *Mesechinus wangi* sp. nov. (Type KIZ 022028) (A), *M. miodon* (Type BM 9.1.1.9) (B), *M. h. hughi* (KIZ 027029) (C), and *M. d. dauuricus* (KIZ 027005) (D)

As *M. miodon* was named based on its small P^3^ (triangular (equal-sided) in shape; Thomas, 1908)), examination of teeth was unavoidable here. *Mesechinus dauuricus* could be diagnosed by P^3^ similar to P^2^ in size; *M. miodon* could be diagnosed by P^3^ smaller than P^2^ (and smaller than that of *M. dauuricus*); *Mesechinus wangi* **sp. nov.** and *M. hughi* could be diagnosed by P^3^ small, though similar to *M. miodon* (Figure 4). All species showed reduced upper M^3^ and small trigonid (Figure 4). Most notably, *Mesechinus wangi* **sp. nov.** could be further distinguished by consistent presence of single-rooted M^4^ on all specimens examined (Figure 5), much smaller than M^3^.

### Morphometric analyses

External and cranial measurements of each species are given in Table 1. Thirty intact skulls were used for PCA, including specimens of *M. dauuricus manchuricus* (*n*=1), *M. hughi hughi* (*n*=20), *M. miodon* (*n*=6), and *Mesechinus wangi* **sp. nov.** (*n*=3).

The first principal component (PC1) accounted for 74.24% of variation (eigenvalue=8.91) and was positively correlated with all variables, reflecting a size effect (Table 2). The second principal component (PC2) accounted for 9.40% of variation (eigenvalue=1.13) and was dominated by MTW (loading=0.93), but was also positively correlated with PL, BM^1^, LBTR, BL, and LUTR (loading>0.53). The third principal component (PC3) represented 4.90% of variation (eigenvalue=0.59) and was correlated primarily with GWN (loading=0.97).

**Table 2:** Factor loading eigenvalues and percentage of variance explained for PC1, PC2, and PC3 from principal component analysis

Variables	Component		
	1	2	3
CH	0.916	0.200	−0.136
CBL	0.836	0.461	0.221
GLS	0.830	0.483	0.160
BL	0.800	0.541	0.141
LUTR	0.720	0.538	0.236
ZMB	0.632	0.450	0.288
IOB	0.542	0.480	0.458
MTW	0.293	0.928	0.026
PL	0.523	0.721	0.292
BM^1^	0.623	0.696	0.216
LBTR	0.631	0.683	0.155
GWN	0.034	0.093	0.970
Eigenvalues	8.909	1.128	0.588
Total variance explained (%)	74.241	9.403	4.899

Abbreviations are given in the Materials and Methods section.

**Figure 6:**
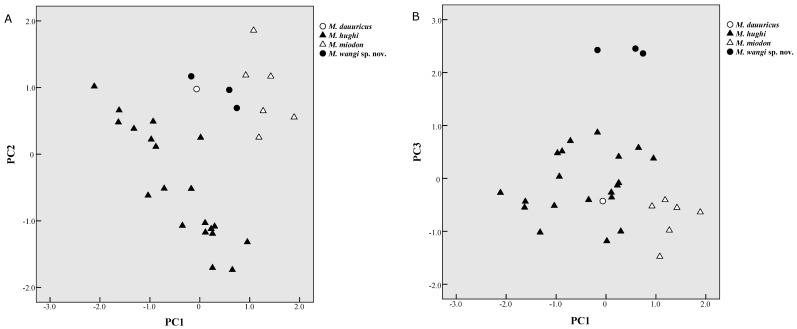
Plot of *Mesechinus* spp. for principal component factors 1 and 2 (A) and 1 and 3 (B)

As shown in Figure 6A, *M. dauuricus*, *M. miodon*, and *Mesechinus wangi* **sp. nov.** plotted closely in the positive region of PC1 and PC2, indicating that these taxa had larger skulls. Further, *M. hughi* plotted in the negative region of PC1, indicating this species had a smaller skull. In the PC1 and PC3 figure (Figure 6B), *Mesechinus wangi* **sp. nov.** plotted in the positive region of PC3 against all other species, indicating this species had the widest nasal.

We employed one-way ANOVA for all external and cranial variables. The results showed that all variables were significantly different among the four species (*P*<0.001), except for HB (*F*=2.080, *P*=0.134), HF (*F*=0.522, *P*=0.596), and PL (*F*=7.561, *P*=0.002).

### Karyotypic characteristics of *Mesechinus wangi* **sp. nov.**

**Figure 7:**
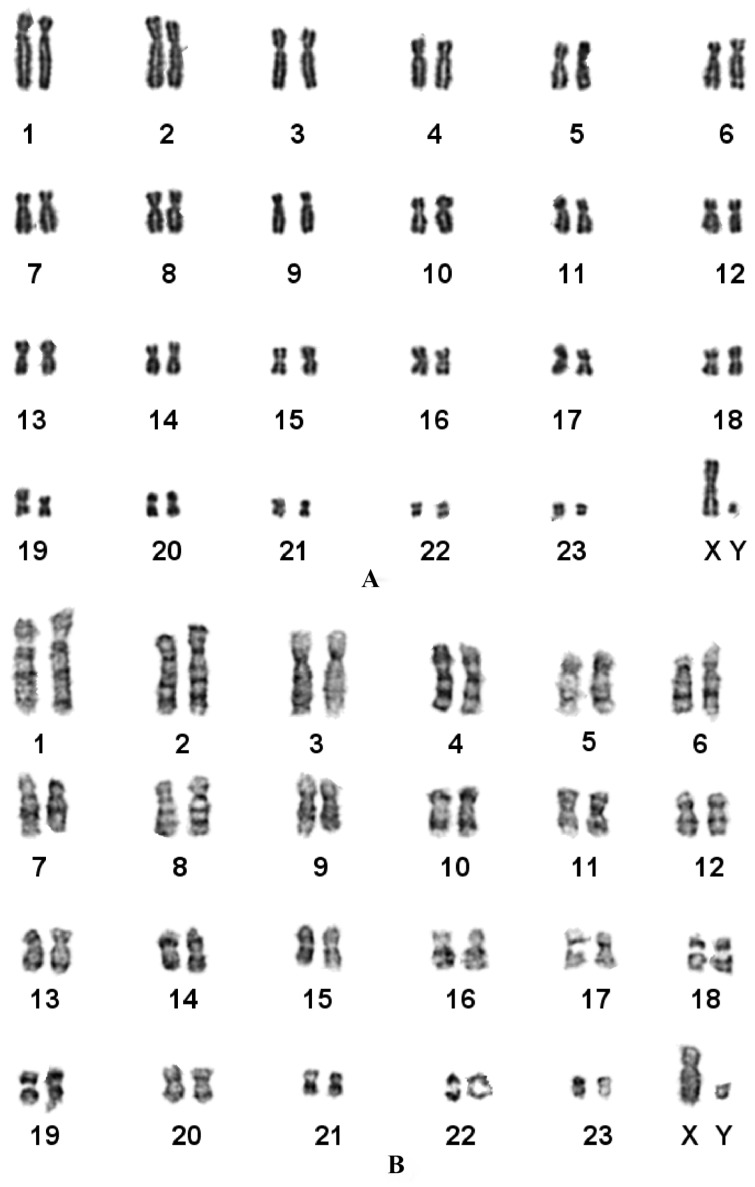
Karyotypes of *Mesechinus* sp. (KIZ 034115)

The karyotypes of *Mesechinus wangi* **sp. nov.** are shown in Figure 7. The diploid number (2*n*) and autosomal fundamental number (FNa) were 48 and 92, respectively (Figure 7A). The autosomes and X chromosomes were biarmed; however, we could not determine whether the Y chromosome was biarmed as it was too small. In total, 22 metacentric + 24 submetacentric autosomes were found in the karyotype. Both the X and Y chromosomes were metacentric, with the Y chromosome being smallest. G-banded karyotypic analysis identified homologous chromosomes (Figure 7B).

A: Conventional karyotype; B: G-banding karyotype.

Compared with other species in the genus *Mesechinus*, *Mesechinus wangi* **sp. nov.** had the same 2*n* and FNa as *M. dauuricus* and *M. hughi*, but differed from the reported karyotype of *M. miodon*, which is characterized by the presence of 0–4 B-chromosomes (2*n*=44–48; FNa=82–92; Table 3). The numbers of metacentric chromosomes (M), submetacentric chromosomes (SM), and subtelocentric chromosomes (ST) also differed among species.

**Table 3:** Karyotypes of the four recognized *Mesechinus* species

Species	2*n*	NFa^1^	Autosomes	X/Y chromosomes	Locality	Reference
*M. dauuricus*	48	92	22M+14SM+10ST	SM/T	Chita, Russia	Korablev et al., 1996
*M. hughi*	48	92	30M+12SM+4ST	M/T	Yulin, China	Lin & Min, 1989
*M. miodon*	44	82	18M+24SM	ST/T	Yulin, China	Lin & Min, 1989^2^
	44–48^3^	84–92	18M+24SM+0–4B(M/SM)	ST/T	Yulin, China	Kong et al., 2016
*M. wangi* **sp. nov.**	48	92	22M+24SM	M/M^4^	Baoshan, China	This study

^1^: Diploid chromosomes classified into metacentric (M), submetacentric (SM), subtelocentric (ST), and telocentric chromosomes (T). ^2^: Animals were identified as *M. dauuricus* in the original article. Kong et al. (2016a) argued that the specimens should be *M. miodon*. ^3^: Because of the existence of 0–4 B-chromosomes (M or SM; Kong et al., 2016a), the 2*n* could be 44–48 and NFa could be 84–92. ^4^: Y chromosome most likely biarmed (see Figure 6B), but was too small to be confirmed.

## DISCUSSION

### Taxonomic implications

We compared the morphology and karyotypes among *Mesechinus* taxa in China. Although sample sizes were small for several forms, the patterns detected in the morphological and karyotypic analyses helped clarify the taxonomy of this genus. It is worth noting, as well discussed in Gould (2001), that dental structures in hedgehogs can exhibit considerable intraspecific variation, and all dental characters should be treated with caution.

*Mesechinus miodon* is still recognized as a subspecies of either *M. dauuricus* (Corbet & Hill, 1992) or *M. hughi* (Hutterer, 2005). Here, however, we recognized *M. miodon* as a distinct species based on morphometric and karyotypic analyses (Figure 6; Table 3). Thomas (1908) named *M. miodon* and *M. hughi* but did not compare either with *M. dauuricus*. In the current study, *M. miodon* was easily distinguished from *M. hughi* (Figure 6A), with its obviously larger cranial measurements (Table 1). *Mesechinus miodon* and *M. dauuricus* exhibited similar overall skull shape and size (Table 1, Figure 6), but *M. miodon* was distinguishable based on different spine color pattern and smaller P^3^.

The implications of the karyotypic evidence are two-fold. On the one hand, *M. miodon* had a smallest number of metacentric chromosomes in the genus, and the numbers of submetacentric and subtelocentric chromosomes were also different from that of *M. dauuricus*, indicating that these two morphologically similar forms were distinct species. On the other hand, the existence of B chromosomes in the topotype of *M. miodon* (from Yulin), as reported in previous studies (Lin & Min, 1989; Kong et al., 2016a), should be treated with caution. Although B chromosomes are heterochromatic and can be verified easily using the C-banding karyotypic approach (e.g., Badenhorst et al., 2009), this was not adopted in the original studies mentioned above. The number of B-chromosomes is usually stable, rather than highly variable as reported for *M. miodon* (0–4), and is usually an odd number, rather than an even number (Table 3) as reported in other mammals (e.g., Badenhorst et al., 2009). Thus, reexamination of the C-banding karyotype using additional samples is warranted. Finally, B-chromosomes are considered adaptive characters that can vary between populations and may be a poor characteristic for distinguishing species.

**Figure 8:**
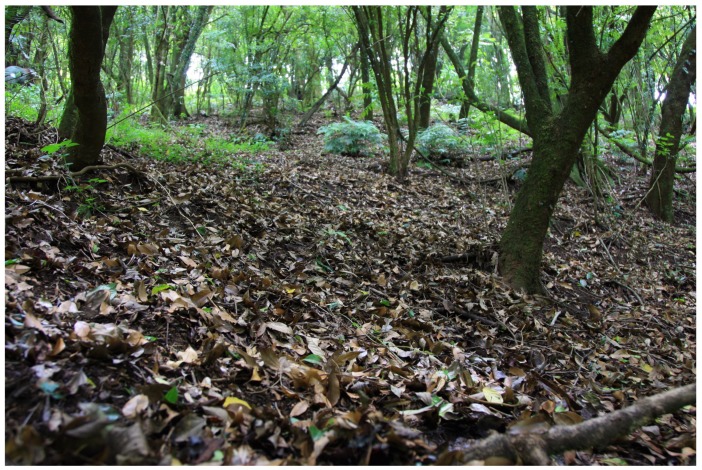
Habitats of *Mesechinus wangi* sp. nov. within Gaoligongshan National Nature Reserve

We recognized the hedgehogs from Mt. Gaoligong as a distinct new species due to their many unique features. This new species is the first known hedgehog to be found at elevations higher than 2 100 m (*Erinaceus europaeus* and *M. hughi* are distributed no higher than 2 100 m), while also inhabiting subtropical evergreen broad-leaved forests (Figure 8) and co-occurring with gymnures. The color pattern of its spine is distinguishable from other *Mesechinus* species due to the lack of a narrow white ring (Figure 3A). It has a broad nasal that shares a long common suture with the maxilla (Figure 4A), which differs from all other taxa. The presence of a supernumerary M^4^ is also highly distinctive (Figure 5A). We propose the name of *Mesechinus wangi* **sp. nov.** for the new species, in memory of the late Prof. Ying-Xiang Wang, a highly respected mammologist from the Kunming Institute of Zoology, Chinese Academy of Sciences.

### Supernumerary upper molar

During the evolution of mammalian dentition, changes in tooth number are very common between taxa and within species, including in the eulipotyphlan mammals. Differences in number of teeth can be used as diagnostic characteristics in shrews (e.g., between *Crocidura* and *Suncus*) and talpid moles (e.g., between *Euroscaptor Mogera*, and *Parascaptor*), especially the number of incisors and premolars (or unicuspids in Soricidae). These differences are often triggered by geographic isolation and speciation, such as observed in the Persian mole (*Talpa davidiana*; Kryštufek et al., 2001) and Japanese mole (*Mogera wogura*; Asahara et al., 2012). Nevertheless, an increased number of molars is an extremely rare event observed in only a few taxa, such as the bat-eared fox (*Otocyon megalotis*), and can be impacted by the evolution of different feeding behaviors and explained using an inhibitory cascade model (Asahara, 2013; Asahara, 2016; Asahara et al., 2016). For example, adaptation toward an increased bite force in the hypercarnivorous bush dog (*Speothos venaticus*) resulted in an enlarged M_1_, thus prohibiting the development of M_3_ dentition (Damasceno et al., 2013). Although erinaceids are characterized by highly variable dentition and tooth structure (Gould, 2001), M_4_ and M^4^ have never been observed in either living or fossil species. Loss of M_3_ has been observed in short-faced hedgehogs, an extinct subfamily of gymnures (Brachyericinae), following remarkable shortening of the skull (Rich & Rich, 1971). Based on teeth features, Lopatin (2006) hypothesized that brachyericines from Asia underwent adaptation toward carnivory. If the inhibitory cascade model is valid in Erinaceidae, the presence of a supernumerary M^4^ in *Mesechinus wangi* **sp. nov.** could be attributed to a combination of genetic bottleneck and isolation (hypothetically after long-distance dispersal from northern China) as well as adaptive selection from an omnivorous-insectivorous diet toward a highly omnivorous one resulting in reduced inhibition during the development of the upper molars (Asahara, 2016; Asahara et al., 2016).

### Taxonomic accounts

#### *Mesechinus dauuricus* Sundevall, 1842 Daurian Hedgehog

*Erinaceus dauuricus* Sundevall, 1842: 237. Type locality: Dauuria, Transbaikalia, USSR.

*Hemiechinus przewalskii* Satunin, 1907: 181. Type locality: North China.

*Hemiechinus manchuricus* Mori, 1927: 108–109. Type locality: “Koshurei, South Manchuria” (=Gongzhuling, Jilin, China).

*Hemiechinus dauuricus* (Sundevall), Satunin, 1907: 185.

*Erinaceus* (*Mesechinus*) *dauuricus* (Sundevall), Ognev, 1951: 8–14.

*Mesechinus dauuricus* (Sundevall), Frost et al., 1991: 30.

Hedgehog of genus *Mesechinus* (GLS=55.18 mm; Table 1). Length of ear similar to surrounding spines. Spines 21–23 mm in length, white for two-thirds of length, followed by black ring, light narrow ring, and black tip (Figure 3); premaxilla extending posteriorly to frontal (Figure 4); P^3^ triangular (equal-sided) in shape, similar to P^2^ in size (Figure 4).

**Distribution**: Widely distributed in eastern Inner Mongolia, Shaanxi, Ningxia, Heilongjiang, Jilin, and Liaoning, China; NE Mongolia; Transbaikalia and upper Amur Basin, Russia (Figure 1).

**Comments**: Because *M. miodon* has been recognized previously as a synonym or subspecies of this species, the distribution boundary between these two species is unclear and the specimens from the southern-most distributions (especially in northwestern China Ningxia and Shaanxi) need to be carefully re-examined. 

#### *Mesechinus hughi* (Thomas, 1908) Hugh’s Forest Hedgehog

*Erinaceus hughi* Thomas, 1908: 966. Type locality: Paochi (=Baoji), Shaanxi, China.

*Hemiechinus sylvaticus* Ma, 1964: 31–36. Type locality: Qin-Shui District, Northern slope of Mt. Lishan, Shanxi, China.

*Hemiechinus dauuricus* (Sundevall), Corbet, 1978: 15.

*Mesechinus hughi* (Thomas), Frost et al., 1991: 30–31 (including *M. sylvaticus*). 

Smallest species of *Mesechinus* (GLS=48.46 mm; Table 1). Ears not longer than surrounding spines; ventral pelage light brown; spines 19–21 mm in length, color pattern same as *M. dauuricus* (Figure 3); frontal relatively higher than parietal; short spine on posterior palatal shelf moderately developed; epipterygoid process moderately developed; basisphenoid moderately inflated; nasal narrow, premaxilla extending posteriorly, frontal extending anteriorly, not meeting premaxilla, nasal and maxilla sharing short suture; P^3^ triangular (nearly equal-sided) in shape, smaller than P^2^.

**Distribution**: Southern Shaanxi, southern Shanxi, and northern Sichuan in China (Figure 1). 

**Comments**: We recognized *H. sylvaticus* (Ma, 1964) as a synonym of *M. hughi*. To date, its taxonomic status has not been appropriately evaluated as it is only known from its holotype. Ma (1964) described *sylvaticus* as a new species but did not examine the specimens of *M. hughi*. Here, the characters used to define *sylvaticus*, such as spine color pattern and presence of sagittal ridge, were observed on all specimens of *M. hughi* examined. Therefore, we recognized *sylvaticus* as a synonym of *M. hughi*. This species inhabits mountainous broad-leaved forest, distinct from *M. dauuricus* and *M. miodon*, and its overall dark color may be an adaptation to such environments.

#### *Mesechinus miodon* (Thomas, 1908) Small-toothed Forest Hedgehog

*Erinaceus miodon* Thomas, 1908: 965. Type locality: Yulinfu (=Yulin), Shaanxi, China.

*Erinaceus europaeus miodon* Thomas, Allen, 1938: 47–54.

*Hemiechinus dauuricus* (Sundevall), Corbet, 1978: 15.

*Hemiechinus dauuricus* (Sundevall), Min & Lin, 1989: 4.

*Mesechinus dauuricus* (Sundevall), Frost et al., 1991: 30.

*Mesechinus miodon* (Thomas), Wang, 2003: 4.

Large species of *Mesechinus* (GLS=54.10 mm; Table 1). Ventral pelage pale white; spines 22–29 mm long, first two-thirds light brown (ivory white in other species), then broadly ringed blackish-brown, terminal 3–4 mm of spine light brown; parietal higher than frontal; well-developed spine on posterior palatal shelf; epipterygoid processes well developed; nasal narrow, premaxilla extending posteriorly, frontal extending anteriorly on dorsal side, touching premaxilla, nasal and maxilla without common suture; P^3^ triangular (nearly equal-sided) in shape, smaller than P^2^.

**Distribution**: Northern Shaanxi and eastern Ningxia, China (Figure 1).

**Comments**: Named as *Erinaceus miodon* based on small P^3^ (Thomas, 1908). Corbet (1978) considered it as a synonym of Hemiechinus dauuricus, which was subsequently followed by many researchers (e.g., Corbet, 1978, 1988; Corbet & Hill, 1992). Hutterer (2005) assigned it as a synonym of *M. hughi* (perhaps following a comment in Frost et al. (1991)). However, both the skull size and shape were very distinct from *M. hughi* in our morphometric analyses (Figure 6). Furthermore, it could be distinguished from *M. dauuricus* based on the different color patterns on the spine (Figure 3) and smaller P^3^. The epipterygoid processes were also longer than those in *M. dauuricus*, though the sample size was small (Figure 4). We therefore recognized it as a valid species.

The karyotype of animals from the type locality varied from 2*n*=44–48 due of the presence of B chromosomes (Lin & Min, 1989; Kong et al., 2016a). Kong et al. (2016b) reported both *M. miodon* and *M. dauuricus* as distributed in Yuling in northern-most Shaanxi; however, the distribution boundary remains unknown. 

#### *Mesechinus wangi* sp. nov. HE, JIANG, and AI

**Common names**: Gaoligong Forest Hedgehog (高黎贡林猬, Gaoligong Linwei) or Wang’s Forest Hedgehog (王氏林猬, Wangshi Linwei)

**Holotype**: KIZ 022028 (field number: 201012001), adult female collected from Gaoligongshan National Nature Reserve (N24°50′, E98°45′), Baoshan, Yunnan, China, on 1 September 2010 at an altitude of 2 215 m a.s.l.. Alcohol-preserved and cleaned skull are deposited in KIZ, CAS. 

**Paratypes**: KIZ 027001 (field number: 0907003), KIZ 027002 (field number: 0907001), KIZ 022027 (field number: 1102007), KIZ 034255 (field number: 201507001), and KIZ 034115 (field number: GLGS 20160601) collected from Gaoligongshan National Nature Reserve, southwestern Yunnan, China from 2003 to 2016 at elevations of 2 100 to 2 680 m. Except for KIZ 034115, which is preserved in fluid, all other specimens are preserved as dried skins with cleaned skulls. The skull of KIZ 027002 is broken and the skull of KIZ 034255 is missing. 

**Etymology**: Named in memory of Prof. Ying-Xiang Wang (1938–2016), head of the mammal research group at the Kunming Institute of Zoology, Chinese Academy of Sciences. He undertook extensive research on the taxonomy, phylogeny, zoogeography, and conservation of mammals and made distinguished contributions to Mammalogy in China (Jiang, 2016). 

**Diagnosis**: Body size larger than *M. hughi*, but similar to *M. dauuricus* and *M. miodon*. Most spines (>80%) lack white ring, in contrast to other *Mesechinus* species. Frontal higher than parietal, differing from *M. dauuricus* and *M. miodon* but similar to *M. hughi*. Spine on posterior palatal shelf short, similar to *M. hughi* but different from *M. dauuricus* and *M. miodon*. Epipterygoid processes longer than that on *M. dauuricus* and *M. hughi*, but shorter than that on *M. miodon*. Basisphenoid uninflated, distinct from other *Mesechinus* species with basisphenoids moderately inflated. Nasal (~4.30 mm) broader than all other *Mesechinus* species (<3.00 mm). P^3^ smaller than that of *M. dauuricus*, but similar to *M. hughi* and *M. miodon*. Supernumerary M^4^ consistently present after each M^3^, unique among all living hedgehogs (Figure 5). 

**Description**: Large *Mesechinus* species (HB=202.40 mm; GLS=54.75 mm; CBL=54.55 mm; Table 1). Absence of spineless area on scalp; length of ear equal to surrounding spines; ventral pelage dark brown; spines 22–25 mm long, most spines (>80%) white for two-thirds of length and black for other one-third, small number of spines (<20%) white for two-thirds of length, then ringed in black, followed by narrow white ring, tip black; frontal higher than parietal; short spine (~1 mm) present on posterior palatal shelf, extending only slightly posteriorly; epipterygoid processes extend labially (2–3 mm); basisphenoids uninflated, two basisphenoids on both sides touch medially, excavated into shallow spherical fossa (namely, nasopharyngeal fossa; see Frost et al., 1991), breadth of nasopharyngeal fossa ~1.5 mm, breadth of nasal ~4.3 mm; premaxilla extending slightly posteriorly and frontal extending slightly anteriorly on dorsal side of skull, not meeting each other, nasal and maxilla sharing suture (~5–8 mm); jugal large, reaching lacrimal, lacrimal/maxilla suture unfused in adults; I^2^ small, I^3^ with two roots, larger than I^2^, P^2^ rectangular shaped, similar to I^3^ in size but larger than C^1^, P^3^ smaller than P^2^, triangular (nearly equal-sided) in shape, M^3^ heavily reduced, hypocone and metacone absent, M^4^ single rooted, much smaller than M^3^ (Figure 5). 

**Measurement**: Measurements for *Mesechinus wangi* **sp. nov.** (KIZ 027028, 027001, 027002, 022027 and 034225) are presented in Table 4.

**Table 4:** External and craniodental measurements for type specimens of *Mesechinus wangi* sp. nov.

Variable	Holotype	Paratypes			
	022028	022027	027001	027002	034255
W	449.00	336.00	390.00	430.00	451.00
HB	240.00	215.00	200.00	180.00	177.00
TL	18.20	18.00	14.00	18.10	18.00
HF	48.00	48.00	45.30	46.70	48.00
EL	31.10	28.90	31.80	28.20	28.00
GLS	55.10	55.60	53.70	54.60	–
CBL	54.80	55.20	53.60	54.60	–
CH	17.50	17.60	16.10	17.30	–
BL	50.50	51.30	50.50	47.70	–
PL	30.80	30.60	30.10	29.50	–
ZMB	34.10	33.70	34.10	–	–
IOB	15.10	14.80	14.20	14.60	–
MTW	26.20	26.20	25.30	24.70	–
GWN	4.30	4.30	4.30	–	–
BM^1^	21.50	21.10	21.70	–	–
LUTR	29.10	28.70	27.10	26.70	–
LBTR	25.30	24.20	25.20	24.70	–

Abbreviations are explained in the Materials and Methods section. –: Not available.

**Comparisons**: *Mesechinus wangi* **sp. nov.** can be characterized by many unique features within *Mesechinus*, including unique color pattern on spine, uninflated basisphenoid, broad nasal, long common suture shared by nasal with maxilla, and presence of M^4^. It is similar to *M. dauuricus* and *M. miodon* in overall size (HB=202.40±26.10 mm) but is obviously larger than *M. hughi* (HB=189.71±24.20 mm; Table 1). *Mesechinus wangi* **sp. nov.** differs from *M. dauuricus* and *M. miodon* in relatively higher frontal than parietal in skull. 

**Distribution**: To date, this species is known only from three counties (Tengchong, Longling, and Longyang) of Baoshan in Yunnan, China, at elevations ranging from 2 200 m–2 680 m. The habitat is subtropical evergreen broad-leaved forest formed by a variety of vegetation, including Fagaceae, Lauraceae, Ericaceae, and Theaceae (Figure 8). 

**Comments**: Population size is currently unknown. However, the known distribution is extremely small and located only within the Gaoligong National Nature Reserve. The species hibernates from middle of October to the following early April.

### Key of four species of *Mesechinus*

1. Ventral pelage dark brown; white for two-thirds and black for other one-third on most spines; greatest width of nasal≥4.00 mm; nasal shares long common suture with maxilla; basisphenoid uninflated; supernumerary M^4^ present after M^3^, occurs only in Mt. Gaoligong, Yunnan, China..…….……...…..…*Mesechinus wangi* **sp. nov.**
  Ventral pelage light brown or white; nasal narrower than 3.00 mm; premaxilla meets frontal on dorsal side of skull (or nearly), nasal does not share suture with maxilla; basisphenoid moderately inflated; M^4^ not present; occurs outside of Yunnan………………………………….…..….2
2. Overall small; GLS<53.00 mm, LUTR<27.00 mm; frontal relatively higher than parietal; occurs in southern Shaanxi, southern Shanxi, and northern Sichuan, China………………….……..………..…*M. hughi*
  Overall large; GLS>53.00 mm, LUTR>27.00 mm; parietal relatively higher than frontal, spine on posterior palatal shelf well developed…………………......….............3
3. Spines 21–23 mm in length; tip of spines black, followed by narrow white ring; epipterygoid processes short; P^3^ similar to P^2^ in size; distributed in northern China, Mongolia, and Russia…………………….….…….....*M. dauuricus*
  Spines 22–29 mm in length; tip of spine light brown; epipterygoid processes well developed; P^3^ obviously smaller than P^2^..….……..………...............*M. miodon*

## Supplementary Material

http://www.zoores.ac.cn/fileup/2095-8137/SUPPL/20180720192924.zip
